# Facile method for 3D printing conformally onto uneven surfaces and its application to face masks

**DOI:** 10.1038/s41598-023-48547-x

**Published:** 2023-12-08

**Authors:** Zehao Ji, Douglas A. J. Brion, Kerr D. G. Samson, Sebastian W. Pattinson

**Affiliations:** Department of Engineering, University of Cambridge, Trumpington Street, Cambridge, CB2 1PZ UK

**Keywords:** Engineering, Biomedical engineering, Mechanical engineering

## Abstract

Conventional additive manufacturing processes, where parts are built through layer-wise deposition of material on a horizontal plane, can be limiting when a part must be printed or fit onto uneven surfaces. Such situations will arise with increasing frequency as additive manufacturing application areas such as construction and medical devices continue to grow. In this work, we develop a simple and practical approach to generate toolpaths to print 3D structures onto uneven surfaces conformally. The algorithm uses only conventional planar toolpaths of both the structure to be printed and the substrate to be printed on and converts these to non-planar toolpaths, allowing easy integration with existing additive manufacturing workflows. The technique is demonstrated by printing flexible seals onto bespoke rigid face mask frames conformally via a conventional single-material 3D printer using the generated conformal toolpath. A notable improvement in air seal performance was observed for customized face masks with conformal soft seals compared to conventionally 3D-printed fully rigid face masks. This also shows the potential of the developed toolpath generation method to aid in the prototyping and fabrication of conformal medical and other devices.

## Introduction

Fused filament fabrication (FFF) is the most widely used additive manufacturing (AM) process for reasons including ease of use, low-cost, and its compatibility with many materials^1,2^. In this process, the path that the nozzle takes to manufacture a part plays an important role in the properties of the final part. Conventional FFF toolpaths consist of sequential horizontal layers. These can affect the mechanical properties of the printed parts because bonding tends to be stronger within a printed filament than between filaments. The toolpath also has a significant effect on the part surface finish because of the stair-step visible on the outer layers of the FFF parts^3^. These and other factors have motivated many studies of 3D FFF toolpath generation where the extruder Z-height can continuously change throughout a print^4–6^. Non-planar printing could improve both the mechanical strength and surface finish of printed parts, as well as reduce material use and printing time, for instance by reducing the need for support material^7–10^. Toolpaths can also run over the surface of a non-planar substrate to fabricate conformal components^11–13^. There have been several implementations of conformal printing for parametric surfaces^8,14,15^. In one example, an analytical surface was divided into an array of data points in a 2D plane by using a rectangular grid with the same dimension as the surface and a spacing equal to the width of the printed filaments^9^. The Z-value of each point on the surface was then calculated to create non-planar printing paths. In another example, the printing trajectories were parameterized onto Bèzier surfaces of arbitrary order to 3D print lattice-shells on non-planar surfaces^14^. However, using these methods can be challenging when needing toolpaths for printing over more complex freeform surfaces such as those produced by 3D scans for biomedical applications.

Conformal toolpaths have also been created on STL mesh surfaces to overcome surface geometry restrictions. In one case, an algorithm projected a planar printing path of sequential and ordered points onto an arbitrary triangle-tessellated surface. A conformal toolpath was then generated by finding the intersection of each projected point of the path and the triangular planes on the tessellated surface^16^. Parametric trajectories, such as the Hilbert curve and re-entrant pattern, were conformally printed on the freeform surfaces. An approach was also developed to generate trajectories to print a 3D tessellated structure conformally onto another tessellated freeform substrate model^17^. However, this approach has the restriction that the bottom surface of the printed structure must match the surface of the freeform substrate. Modifications to the CAD design are required to fill the free spaces between the printing structure and the substrate if there is any misfit.

In this work, we developed a practical approach for generating conformal toolpaths to enable the fabrication of 3D structures onto freeform substrates without parameterizing the substrate surface or modifying the geometric design of printing structures. In order to show the potential of adopting this approach to manufacture conformal medical devices with improved efficacy, in this work conformal 3D printing of user-customized respirators was demonstrated.

SARS-CoV-2, the virus that led to coronavirus disease 2019 (COVID-19), spreads from person to person through aerosols and droplets of respiratory fluids produced by an infected person while coughing, talking, sneezing, or breathing^18,19^. To reduce or interrupt SARS-CoV-2 transmission, public health organizations suggested that the public wear respiratory personal protection equipment (PPE)^20,21^. In addition to filtration efficiency, the effectiveness of respirators is highly dependent on how well the respirator fits the individual facial profile of the wearer. Inappropriate fitting of the respirator would lead to gaps or leaks between the user’s face and the respirator, increasing the risk of infection during COVID-19^22,23^. Commercially available respirators are typically designed based on average facial features to fit the widest range of users; however, it is challenging to develop a device that fits everyone perfectly^24,25^. For example, previous studies have highlighted lower pass rates of respirator fit tests for females compared to male participants^26,27^. This has led to the demand to develop bespoke respiratory devices. Combined with 3D scanning technology, AM has been used to fabricate wearable medical devices that better fit wearers^28,29^. Distributed manufacturing of personalized respirator components via FFF was used to help combat shortages of respiratory devices during the COVID-19 pandemic^30^. Significant improvements in fit and seal performance were observed for FFF-printed personalized respirators compared to surgical and FFP2/N95 face masks^31,32^. Reduction in contact pressure on a user’s face through the use of customized 3D printed face masks, also improving wearer comfort, has also been reported^33^. However, rigid respirator designs can lead to higher contact loads while wearers move their faces^33^ and cause skin damage after prolonged use time^34^. 3D printed rigid face masks with soft seals on the contour of the masks were designed and have been shown to have effects on both reducing wear discomfort and improving air seal performance by providing better compliance with the user’s face^35–37^. However, the manual processing required to fabricate the soft seals and assemble them onto the 3D-printed face mask frames can be time-consuming and costly. Additionally, human error during assembly could cause gaps between the seal and the mask frame.

In this study, flexible seals were conformally printed on the rigid frame of the 3D-printed bespoke face mask using the developed conformal toolpath generation approach and a low-cost single-material FFF 3D printer. Generation of the conformal toolpath simply requires the conventional planar toolpaths for both the printing object and the substrate as inputs, also enabling an easy way to set the desired printing parameters, including infill pattern and density, nozzle size, layer thickness, etc. The improvement in the efficacy of the face mask with a conformally printed soft seal was validated by conducting an air seal performance test on it. Meanwhile, the proposed easy-to-use conformal printing methodology could be potentially implemented for the fabrication of other functional conformal components, such as conformal tactile sensors^38,39^ and other wearable devices, due to its ability to generate toolpaths over freeform surfaces, for example, the contour of the human body which is usually non-parametric surface.

## Methods

### Conformal 3D printing toolpath generation

**Figure 1:**
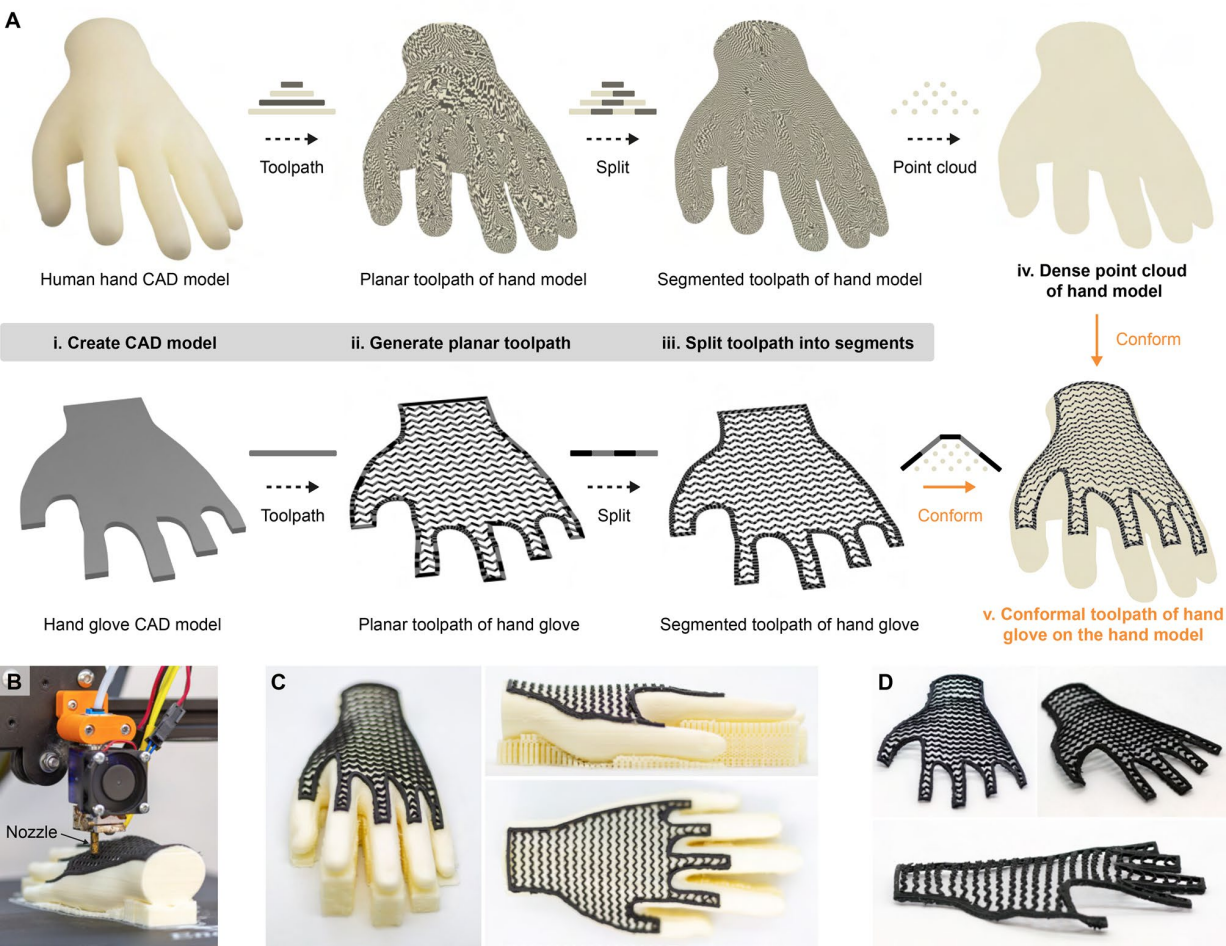
Overview of the conformal toolpath generation process, printing equipment, and conformally printed parts. **(A)** Schematic illustration of steps utilized for generating conformal printing toolpath. **(B)** Optical image of the Creality CR-20 Pro FFF printer used for the conformal printing process. **(C)** Optical images of the hand substrate after conformal printing of the hand glove on it. **(D)** Optical image of the conformally printed hand glove after removing it from the hand substrate.

To explain the approach developed in this work for implementing the conformal printing process, a demonstration of printing a hand glove conformally onto a human hand substrate to fabricate a user form-fitting glove is presented. The workflow used for generating the conformal printing toolpath in this example is illustrated in Fig. 1A.

An existing CAD model of the human hand was downloaded from an online 3D model sharing platform, GrabCAD (GrabCAD, Inc.). The hand glove model was designed using the CAD software Fusion 360 (Autodesk, Inc.) by projecting the human hand model onto a 2D plane. The projected profile was then extruded with a 2mm thickness, which matched the desired thickness of the conformally printed hand glove (Fig. 1Ai). Subsequently, both CAD models were converted to STL files and then sliced into planar printing toolpaths (Fig. 1Aii) by adopting commercial 3D printing slicing software, Simplify3D (Simplify3D, Inc.). A default infill pattern, Wiggle, was selected to slice the hand glove model at 15% infill density. The obtained planar toolpaths of both the hand glove and hand substrate were output in the format of Gcode.

To create the conformal toolpath of the hand glove, the Gcode files of both the hand glove and the hand substrate were input into the conformal toolpath generation main algorithm (Algorithm 1) developed in this study. In Algorithm 1, the Gcode files were processed with four steps to eventually output the toolpath for conformal printing of the hand glove on the surface of the hand substrate.

**Algorithm 1:**
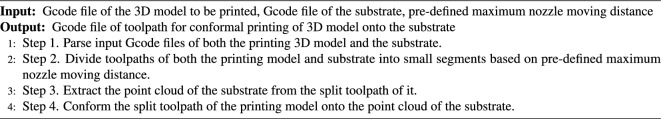
Main algorithm.

In step 1, both Gcode files were parsed individually to extract all Gcode commands in the toolpath. The Gcode commands of the nozzle moving lines (including both the printing lines and the travel lines) were subsequently split into code and argument. Afterward, the commands were categorized based on layers of print, and their functionalities were executed to create a virtual print model (Algorithm 2).

**Algorithm 2:**
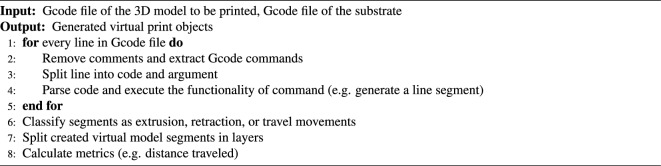
Step 1: parse the input Gcode files.

Once the Gcode files were parsed, the nozzle moving lines in every layer of the virtual print objects were split into small segments (Fig. 1Aiii) based on a pre-defined maximum nozzle moving distance ($d_{max}$) using Algorithm 3. In this demonstration, a maximum distance of 1.0 mm was utilized.

**Algorithm 3:**
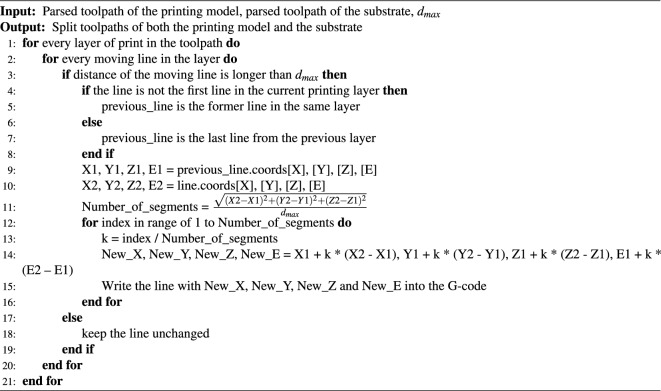
Step 2: divide the toolpaths of printing model and substrate into small segments.

After the print models were split into 1.0mm sections, the printing lines in the split toolpath of the hand substrate were identified from all moving lines. A dense point cloud of the hand substrate model was then extracted from these segmented printing lines (Algorithm 4) by recording the X, Y, and Z coordinate values of the printing lines (Fig. 1Aiv).

Once Algorithm 4 was completed, the split toolpath of the printed glove was conformed onto the output dense point cloud of the hand substrate to eventually generate the conformal printing toolpath (Fig. 1Av) via Algorithm 5. In Algorithm 5, the split toolpath of the hand glove was first parsed using Algorithm 2. Afterward, the X–Y coordinate of every moving line in each layer of the print model was compared with the X–Y coordinates of points in the dense point cloud of the hand substrate. A moving line was regarded as intersecting with the point cloud if the distance difference in both X and Y directions between the line and a point in the point cloud is less than half of the pre-defined maximum nozzle moving distance, and the Z coordinate of the point in the point cloud was recorded in a list (Z_list). This process was repeated until the X–Y coordinate of the moving line was compared with that of every point in the point cloud of the substrate. The maximum Z-height of the point cloud at the X–Y coordinate of the intersection point was identified by finding the maximum value in the Z_list. Afterward, the Z coordinate of the moving line was changed by adding the current Z coordinate value of it with the value of the determined maximum Z-height. If the current processing moving line was a printing line, its extrusion value was also adapted accordingly to compensate for the increased nozzle movement distance due to the change in the Z coordinate, while a line would remain unchanged if it did not intersect with the point cloud. This process was repeated until every moving line in every print layer of the hand glove toolpath had been processed. All the processed lines were stored and eventually output as a toolpath in the format of Gcode. The code used to generate conformal toolpaths in the paper can be found in the public GitHub repository (https://github.com/cam-cambridge/Conformal-3D-Printing).

**Algorithm 4:**
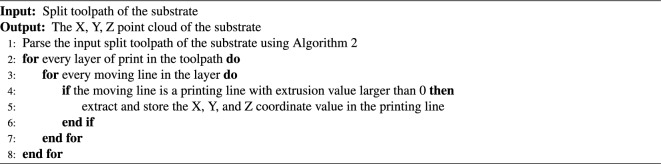
Step 3: extract point cloud of the substrate from toolpath.

**Algorithm 5:**
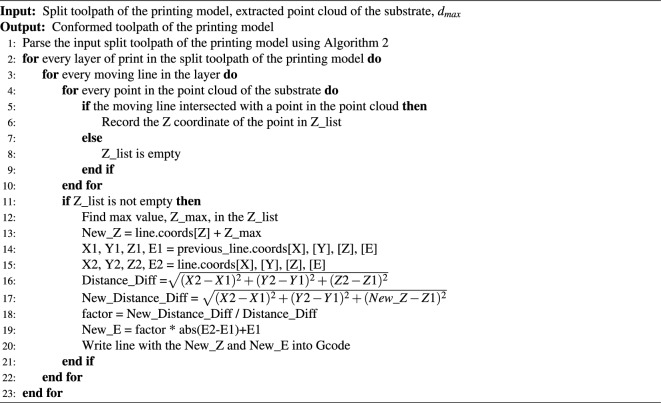
Step 4: conform the split toolpath of the printing model on the dense point cloud of the substrate.

### Conformal 3D printing system

A Creality CR-20 Pro FFF 3D printer was utilized for the conformal 3D printing process. The hot end and nozzle (0.4mm) of the printer were upgraded to E3D Volcano parts. The nozzle was machined on a lathe to steepen the external cone angle of the nozzle tip to 60$^\circ$. An image of the modified hot end and nozzle is presented in Fig. 1B, and the image of the CR-20 Pro 3D printer after modification is illustrated in Supplementary Fig. S1 online.

### Conformal 3D printing of the hand glove

To perform the conformal 3D printing process shown in Fig. 1, the hand substrate was first printed using polyvinyl alcohol (PVA) filament by conventional planar printing with a layer height of 0.2mm. Once the print was complete, the stepper motors on the XYZ linear axes of the printer were locked using the Gcode instruction to maintain the positions of the print bed and the extruder. Subsequently, the PVA filament equipped on the printer was manually changed to a spool of black thermoplastic polyurethane (TPU) filament. The hand glove was eventually printed conformally onto the pre-printed hand substrate using the generated conformal printing toolpath. Optical images showing the conformally printed hand glove before and after dissolving the PVA hand substrate can be seen in Fig. 1C, D, respectively.

### Design and conformal toolpath generation of user-bespoke face mask

**Figure 2:**
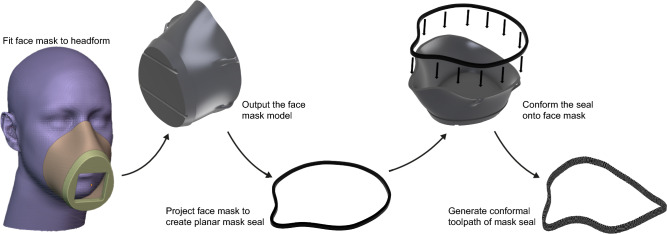
Schematic illustration of the 5-step workflow adopted for designing the customized face mask model and generating the conformal printing toolpath of the mask seal..

A 5-step workflow used to design a user-bespoke face mask model and generate the conformal 3D printing toolpath of the seal of the face mask is visualized in Fig. 2. To design the personalized face mask, a digital headform model was downloaded from the National Institute for Occupational Safety and Health (NIOSH) to use as a reference face of the user^40^. NIOSH conducted a nationwide anthropometric survey of 3997 subjects in 2003, and the gathered data was adopted to develop five headform models in different size categories. These headform models have been used in existing research and have been incorporated into a technical specification standard for SC15 Respiratory Protective Devices. In this study, a medium-sized NIOSH headform model was selected to use as an example.

The generation of the user-bespoke face mask model was implemented using an add-on developed by WASP in Blender 2.82^41^. First, the headform model was imported into Blender, and the face of the headform was oriented in the negative Y direction of the global reference system. Afterward, the border of the mask was manually defined on the facial profile and was subsequently used to create a customized face mask model which followed the pre-defined border. Eventually, the designed face mask model was output in the format of STL, which was imported into Fusion 360 for further operations.

The filter opening of the face mask model was filled by extrusion in Fusion 360 for the purpose of performing subsequent air seal performance tests. To generate a toolpath for conformally printing a mask seal on the top surface of the mask that would intersect the user’s face, the mask was first projected onto a 2D plane to create a 3mm thickness planar mask seal model. Subsequently, the conformal printing toolpath of the mask seal was developed through the same methodology utilized in the hand glove demonstration.

### Manufacture of the face mask prototypes

**Figure 3:**
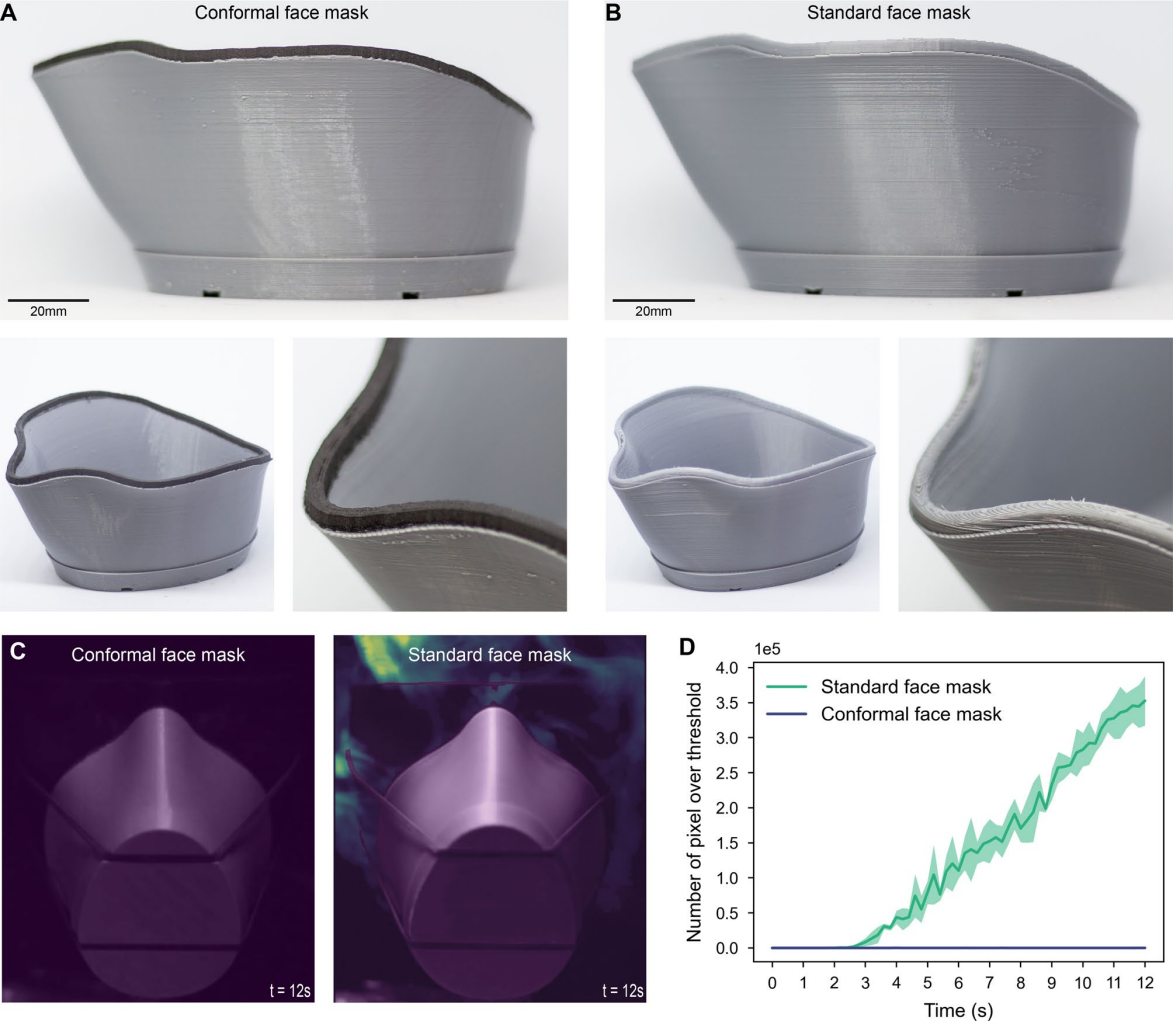
3D printed face mask models and air seal performance test results of them. **(A)** Optical images of the planarly printed mask substrate (grey) with a conformally printed TPU mask seal (black). **(B)** Optical images of fully planar-printed face mask. **(C)** Colormap images for visualizing vapor escaped from face masks in air seal performance tests. **(D)** Plot showing average vapor pixel count against time of three groups of conformal and standard face mask trials.

The modified CR-20 Pro conformal printing system was used to fabricate the designed user-bespoke face mask with a flexible conformal mask seal. Polylactic acid (PLA) filament was chosen for planar 3D printing of the rigid face mask substrate with a layer height of 0.2mm due to its low-cost, ease of printing, and good resistance to chemical disinfectants such as ethanol^42^. Subsequently, conformal 3D printing of the mask seal was performed using black TPU filament at a nozzle temperature of 220 $^\circ$C, print bed temperature of 65 $^\circ$C, a printing speed of 15 mm/s, and a layer height of 0.2 mm. Optical images showing the 3D-printed face mask with a flexible seal (conformal face mask) can be seen in Fig. 3A.

To validate the level of protection solely enhanced by the conformally printed TPU flexible seal on the face mask frame, a standard face mask that had the same geometry as the conformal face mask was also fabricated using PLA through the conventional planar printing approach at a layer height of 0.2mm. Optical images of the planarly printed standard face mask are presented in Fig. 3B.

### Air seal performance test of face mask prototypes

An air seal performance test was performed via vapor flow visualization to evaluate and compare the fit and protection level of the fabricated conformal face masks and standard face masks. An optical image showing the experimental setup is presented in Supplementary Fig. S2 online.

The medium-sized NIOSH headform model was chosen as the facial profile for the air seal performance test. The headform was 3D printed using black PLA filament at 0.1mm layer height and was subsequently placed on a stand within a fume hood, which was covered with black backdrops. Illumination was provided by an LED strip that was placed above the facial profile. A flexible tube with a 10mm inner diameter for vapor injection was connected to the rear of the headform and was approximately located at the mouth position of the facial profile. The face mask prototypes were fastened onto the headform using two elastic bands, and two slots can be found on both the front of the face masks and the rear of the headform model to ensure the identical relative position of the face mask to the headform in each test. A similar test rig was adopted to assess the fit of the face mask in a study by Carter et al.^37^.

In air seal performance tests, vapor was produced via a fog machine (MARQ Fog 400 W) and was delivered into the face mask through the flexible tube. Once the mask was fully filled, vapor leakage might be observed from the places of poor fit that had gaps between the mask and the facial profile. The test processes were recorded with a digital camera (EOS 6D, Canon) at 25 fps, a shutter speed of 0.004 s, and an ISO setting of 1250. For each face mask test, a recording was started approximately 2 s before vapor injection and continued for around 10 s. Three standard and three conformal face masks were manufactured and tested to show the repeatability of the process.

Frames of the recorded videos were post-processed using a bespoke Python script for semi-quantitative evaluation of the seal performance of the face masks. First, a background image was created by averaging grayscale values of the first fifty frames in the video. Subsequently, every five frames of the original footage were averaged, and the averaged images created were subtracted from the background image. A threshold value of 10 was applied and pixels with an absolute deviation higher than the threshold were identified as vapor that escaped from the mask. The vapor pixels were visualized by applying colormap, which was subsequently superimposed onto the background image to approximately present areas of leakage on the mask and the qualitative intensity of the escaped vapor. Example processed images showing vapor leakage for conformal face masks and standard face masks after a vapor injection of 10 s are illustrated in Fig. 3C. The number of vapor pixels was also counted for each processed image to provide a semi-quantitative measurement of the amount of vapor leakage against time.

## Results and discussion

Typically, the use of soft seals on 3D-printed face masks would require additional manual fabrication and assembly processes. It is also challenging to print the seal directly on the mask frame via planar printing using a single-material FFF 3D printer (an example print is shown in Supplementary Fig. S3 online). Using the conformal toolpath generation and printing workflow proposed in this study, a soft seal was successfully 3D printed on the planarly printed user-bespoke face mask frame, avoiding the need for manual procedures and the use of an expensive multi-material 3D printer, thus minimizing potential human error as well as time and cost.

The effect of the conformally printed soft seal on the protection level of the face mask was assessed by performing an air seal performance test. As presented in Fig. 3C, an extensive amount of vapor leaked from the nasal and side cheek areas of the face profile was noticed for the conventional rigid 3D-printed face mask (standard face mask). No obvious vapor escape was observed for the conformal face mask, indicating enhanced air seal performance and compliance with the user’s face provided by the conformally printed soft mask seal. Example animations presenting vapor leakage of the standard and conformal face mask are shown in Supplementary Videos 1 and 2 online, respectively. Repeatability tests were conducted for a total of three groups of conformal and standard face masks. The mean and standard deviation of the vapor loss measurements for the tested face masks are plotted in Fig. 3D. In the plot, the vapor was injected at t=2s and continued until t = 12 s. The measurements validated the rapid and huge amount of vapor leakage for 3D-printed rigid personalized face masks compared to face masks with the additional conformally printed soft seal. These results matched the statements of previous studies that the use of an additional flexible seal would improve the fit rate of a 3D-printed respirator and thus its air seal performance^25,37^.

Although the air seal performance of the masks was effectively assessed, there were limitations to the experimental setup. A fully rigid 3D-printed head mannequin was used as the facial profile in the test. However, the mannequin was unable to replicate compressible skin and varied thicknesses of different soft tissues on the human face. Meanwhile, a fog machine was adopted to continuously pump vapor into face masks during tests, while a linear motor-actuated piston pump could be used instead to mimic a human-breathing-like airflow cycle for vapor delivery. The purpose of this study was to provide a practical and less labor-intensive approach to applying a flexible seal to conventional 3D-printed personalized face masks for improved protection and wearing comfort. Therefore, the tests conducted in this work were intended to compare the performance between 3D-printed face masks with and without the conformally printed soft seal, rather than to evaluate their performance under real-world conditions.

The algorithm and the printing workflow developed in this work have also shown the potential to fabricate other conformal components. As presented in Fig. 1, a hand glove was conformally printed onto a human hand model, and the printed glove still maintained its shape after the PVA hand substrate was dissolved. This demonstrates the ability of the proposed approach to generate a toolpath for conformal printing of a 3D structure onto a freeform surface. The approach could be particularly useful for conformal printing user form-fitting wearable devices, as the human body contour is typically obtained from 3D scanning and is not parametric.

**Figure 4:**
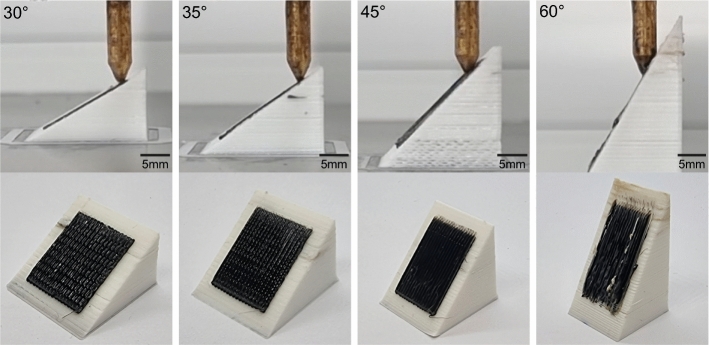
Optical images showing the nozzle when printing along inclined substrates with slope angles of 30$^\circ$, 35$^\circ$, 45$^\circ$, and 60$^\circ$, and the respective states of the substrates after conformal printing. All the substrates have a base area of 20 mm by 20 mm, and the black thin films for conformal printing have a dimension of 15 mm by 15 mm by 0.4 mm.

Challenges also appeared to implement the conformal 3D printing process. First, an E3D Volcano nozzle with a sharpened nozzle tip is required for the printing system. Even using this, the maximum inclination angle that the nozzle can print is limited. This is shown in Fig. 4 where substrates with different inclines ranging from 30$^\circ$ to 60$^\circ$ are printed on. As presented in Fig. 4, printing could be successfully conducted on the substrate inclined up to 35$^\circ$, with no collision between the nozzle and the substrate. However, at a slope of 45$^\circ$, minor surface scratches were noticed on the substrate. The substrate suffered more severe damage when attempting to print at an inclination of 60$^\circ$. The maximum angle between the nozzle and the substrate therefore had to be limited to 35$^\circ$ to ensure collision-free printing. To overcome this issue, previous research has employed multi-axis systems, including 5-axis CNC machines^43^ and 3D printers^44,45^, and 6 DOF robotic arms^46,47^, to perform conformal 3D printing processes. The additional degrees of freedom can help avoid collision between the printing nozzle and the substrate^5^. For instance, Zhang et al.^48^ proposed a novel general slicing method for multi-axis 3D printing by computing the rotation-driven deformation to eliminate supporting structures and improve strength and surface quality. However, the robotic arm utilized in this study may be difficult to access for many users. Alternatively, Hong et al.^44^ have presented an accessible method for upgrading a commercial 3D printer to a 5-axis printer along with a GUI-based conformal slicer running within the Rhino CAD package. Although these studies permit printing on substrates with larger inclination angles, the primary goal of the algorithm presented in this work is to provide an easy-to-use approach for the public to perform conformal 3D printing on freeform substrates using conventional 3D printers and slicers.

A further limitation is that the computational resources needed to generate conformal toolpaths using our method increase with larger part dimensions and shorter line-splitting distances. A potential improvement would be to extract the outer surface of the dense point cloud of the substrate structure instead of the entire dense point cloud for comparison with the split printing lines in the printing structure. Furthermore, there is also a scope to extend the use of the algorithm to the direct ink writing (DIW) approach to conformally print functional inks, including polymers and conductive materials, enabling a wider range of applications, such as wearable biosensors for human motion monitoring and conformal flexible sensors for robotic manipulation.

## Conclusion

This paper presents a simple and practical approach to generating non-planar toolpaths to conformally print 3D structures onto substrates with freeform surfaces. The inputs of the toolpath generation algorithm are the planar toolpaths for both the structure to be printed and the substrate to be printed on created using conventional 3D printing slicing software. This provides a simple way for users to set the desired printing parameters, such as infill pattern, layer height, and extrusion width, and makes it well-suited to the existing 3D printing workflow. Using the developed conformal printing workflow, personalized face masks with attached flexible TPU mask seals were fabricated. The flexible seal was directly printed onto a 3D-printed PLA face mask frame via conformal printing, avoiding manual processes needed for seal fabrication and assembly, thus also minimizing potential human error and reducing time and cost. Vapor flow visualization suggested a notable improvement in the air seal performance of face masks with conformally printed soft seals compared to conventional customized PLA masks because of the improved conformance with the model facial profile provided by the flexible TPU interface material. An inclination angle of 35$^\circ$ was identified as the maximum angle between the nozzle and the substrate for a collision-free print due to the limited mobility of the adopted three-axis Cartesian 3D printing system. Although the maximum angle of inclination is limited, the algorithm and printing workflow proposed in this work could contribute to the community by providing an easy-to-use method for performing conformal 3D printing using an accessible low-cost FFF 3D printer and planar toolpath slicers. Extending beyond face mask fabrication, the algorithm could also be utilized to fabricate other conformal components, particularly user form-fitting wearable devices.

### Supplementary Information

Supplementary Information.

Supplementary Video 1.

Supplementary Video 2.

Supplementary Video 3.

Supplementary Video 4.

## Data Availability

The datasets generated during and/or analyzed during the current study are available from the corresponding author on reasonable request.
