# Supplementary material for: Facile method for 3D printing conformally onto uneven surfaces and its application to face masks
Source: Sci Rep. 2023 Dec 8;13:21659. doi: 10.1038/s41598-023-48547-x (PMC10709438; doi:10.1038/s41598-023-48547-x)
Supplement: Supplementary file 1 — Supplementary Information. [file 41598_2023_48547_MOESM1_ESM.pdf]

Supplementary Information for the manuscript titled:

Facile Method for 3D Printing Conformally onto Uneven Surfaces and its Application to Face Masks

Zehao Ji[1], Douglas A. J. Brion[1], Kerr D. G. Samson[1], and Sebastian W. Pattinson[1,*]

[1]Department of Engineering, University of Cambridge, Trumpington Street, Cambridge, CB2 1PZ, UK
[*]swp29@cam.ac.uk

Source code

Full source code can be found on the CAM group GitHub page: https://github.com/cam-cambridge/Conformal-3D-Printing

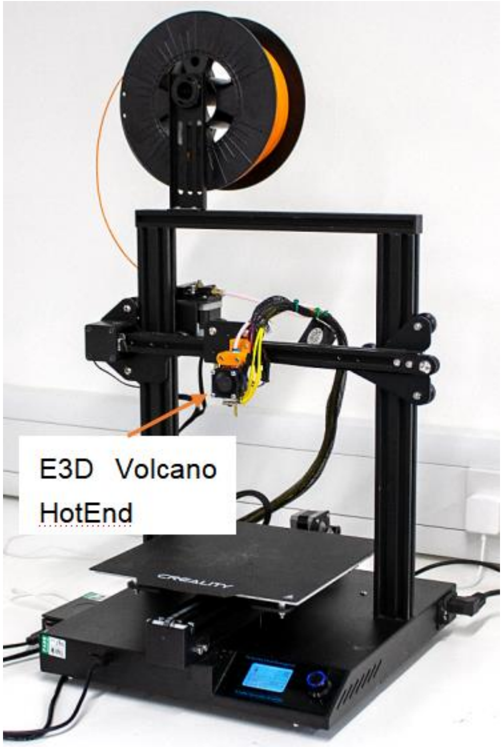

Figure S1: Creality CR-20 Pro 3D printer used for the conformal 3D printing process with upgraded E3D Volcano hotend and nozzle.

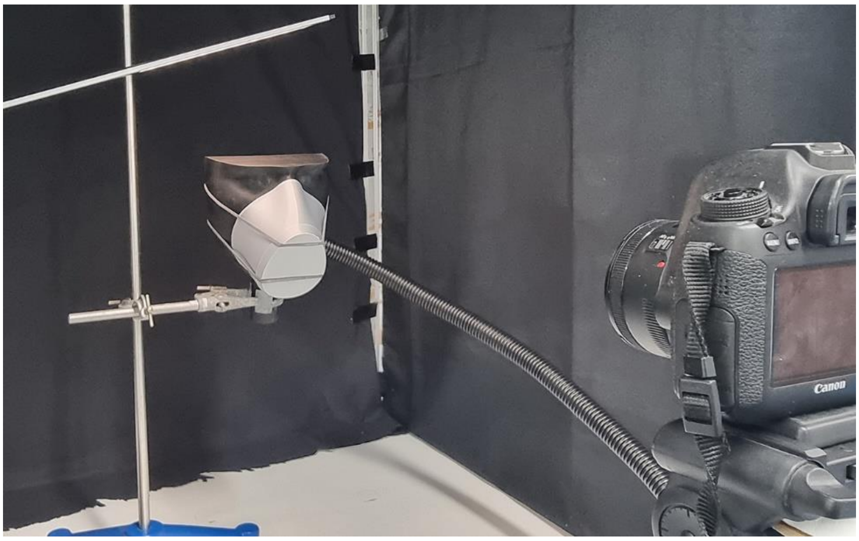

Figure S2: Optical image of the test rig for implementing air seal performance tests of the face masks.

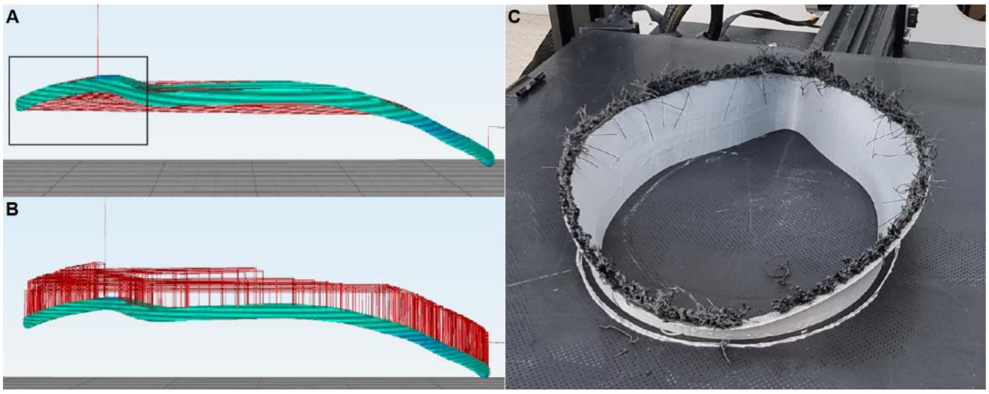

Figure S3: Experiment for attempting the fabrication of the flexible mask seal onto a 3D-printed face mask frame via planar 3D printing. (A) Conventional planar toolpath for printing the face mask seal, resulting in nozzle collision with the substrate. **(B)** Modified planar toolpath for printing the face mask seal with the use of retraction vertical lift to avoid nozzle collision. **(C)** Optical image of the flexible mask seal fabricated using the modified planar toolpath.

In this work, an attempt was made to fabricate the flexible mask seal onto a 3D-printed face mask frame via a more conventional planar 3D printing approach. To conduct the experiment, a 3D model of the mask seal was first created by converting the conformal printing toolpath of the mask seal used in the paper to a dense point cloud and subsequently to the STL file. The created mask seal model would have a 3D structure that fully conforms to the top surface of the face mask frame. Afterward, the STL file of the created mask seal model was imported into a conventional 3D printing slicer, Simplify3D, to generate the conventional toolpath for planar printing.

The generated toolpath is illustrated in Fig. S3 A, where the printed lines and travel lines are green and red, respectively. However, nozzle collision with the face mask frame substrate could happen during nozzle travels within one layer of a print. An example of potential nozzle collisions while traveling is presented in the black box in Fig. S3 A. To avoid the collision, the conventional planar toolpath was modified by using the “retraction vertical lift” function in the Simplify3D software used for toolpath planning, as shown in Fig. S3 B. The nozzle was lifted by a 10mm distance when performing filament retraction and nozzle traveling movements. By using the modified planar toolpath, the face mask seal model was printed using TPU material onto the face mask frame with no obvious nozzle-substrate collision. An optical image of the fabricated TPU mask seal on the 3D-printed face mask frame is shown in Fig. S3 C. Although a filament retraction distance of 2mm with a retraction speed of 20mm/s was applied, molten TPU material still flowed out of the printing nozzle and caused stringing on the printed surface when the nozzle was lifting. In addition, because of the frequent nozzle lifting and travel movements, some areas on the face mask frame did not have material deposited on them, and the surface of the planar printed mask seal was much coarser compared to the seals that were conformally printed in this work. Therefore, without using the conformal toolpath generation approach developed in this study, it could be challenging and time-consuming for users to modify the design of the printed structure so that it has the geometry fully conformed to the substrate, as well as to determine the optimized planar printing parameters to achieve a defect-free print.
